# Using SimulATe to model the effects of antibiotic selective pressure on the dynamics of pathogenic bacterial populations

**DOI:** 10.1093/biomethods/bpz004

**Published:** 2019-05-29

**Authors:** Pedro H C David, Xana Sá-Pinto, Teresa Nogueira

**Affiliations:** 1 Centro de Ecologia, Evolução e Alterações Ambientais (CE3c), Faculdade de Ciências da Universidade de Lisboa, 1749-016 Lisboa, Portugal; 2 Centro de Investigação em Didáctica e Tecnologia na Formação de Formadores (CIDTFF), Universidade de Aveiro (UA), Campus Universitário, 3810-193 Aveiro, Portugal; 3 P.PORTO—Escola Superior de Educação (ESE), Politécnico do Porto, Rua Dr. Roberto Frias 602, 4200-465 Porto, Portugal

**Keywords:** antibiotic resistance, bacterial infection, teaching evolution

## Abstract

Antibiotics are notable weapons in fighting bacteria. Nowadays, however, the effectiveness of antibiotics is severely hindered by the increasing levels of antibiotic resistances in pathogenic bacterial populations, which can persist due to the selective pressure caused by antibiotic exposure. Arguably, the main cause of antibiotic resistances endurance in nature is antibiotic misuse, such as via overusing, inappropriate prescribing as well as the uncontrolled use in agriculture and livestock. There is also a lack of knowledge on appropriate antibiotic usage by the general public. Public scientific literacy and more research on therapeutic practices are fundamental to tackle this problem. Here, we present SimulATe a software which allows the simulation of the effects of antibiotic therapies on bacterial populations during human infections. This software can be used to develop students’ scientific literacy, using infections and antibiotic treatments as context to engage students in scientific practices, and discussions on antibiotic treatment onset and duration or on its use in immunosuppressed or critically ill individuals. SimulATe’s features also allow it to be used for research purposes allowing the simulation of real scenarios and exploration of their outcomes across the parameters’ landscape.

## Introduction

Antibiotics can be crucial for the survival of severe bacterial infections, and its introduction into medical practice led to a decrease in the rate of human death due to infection [1]. In the past decades, antibiotics have been widely and intensively used, both in medical and veterinary practices as well as in the farming and livestock industries [2]. Antibiotic exposure plays an important role in the evolution of microorganisms and shapes both the resistance profile of microbiomes—natural microbial communities—as well as microbial diversity [3]. According to the European Centre for Disease Prevention and Control (ECDC), health workers have been enrolled in public literacy on infection prevention and control as well as on the adequate prescription and dispensing of antibiotics [4, 5]. Yet, despite all the efforts, a survey conducted in 28 European countries reveals that 57% of the Europeans still do not know that antibiotics are ineffective against viruses and 16% had taken antibiotics to treat flu [6]. Incorrect and irresponsible use of antibiotics reduces its effectiveness and compromises the control over bacterial infections, as bacterial communities become resistant to antibiotics. Some of the recommendations of the ECDC towards the reduction of the spreading of antibiotic-resistant bacteria include: reducing antibiotic prescription; timely, dose and duration appropriate administration, in particular in critically ill patients; avoiding early termination and delayed start of antibiotic therapy; and choosing a suitable antibiotic spectrum according to the infecting bacteria [7]. These guidelines aim at preventing antibiotic misuse; nevertheless, treatment of bacterial infections depends on many different variables, such as the dynamics of the microbial community exposed to the antibiotic therapy; microbial diversity; initial antibiotic resistance level; and efficacy of the infected host’s immune system [8, 9]. In opposition to ECDC’s recommendations, recent studies have suggested that interrupting antibiotic therapy as soon as symptoms disappear could be more effective in preventing the spreading of antibiotic resistance [10–13]. These results have recently been advertised in many countries in several newspapers [14–16]. This conflicting information may result in public confusion and mistrust in health care providers’ recommendations further enhancing the problem of antibiotic misuse. In this scenario, a scientifically proficient public, as defined by the US National Research Council (NRC) [17, 18], should be able to understand these results and recommendations and the contradiction with ECDC’s recommendations as part of the scientific knowledge construction. For that, besides understanding what antibiotics are and their effects on bacterial communities, the public also needs to understand how scientific knowledge is produced, to be able to evaluate scientific evidence and to use scientific knowledge to perform informed choices and participate in public debates. In order to achieve this, educational resources that allow the exploration of these four branches of scientific literacy [17], within the context of antibiotic use, are highly needed. Software that model the effect of antibiotic administration under distinct scenarios is of particular interest as educational resources for these purposes as these may allow students to learn about the impacts of antibiotics in bacterial communities, while engaging in scientific practices and discussions that are important for the development of their scientific literacy [17, 18].

Some applications have been developed with the aim of simulating the effect of antibiotic administration, although not completely customizable in terms of parameters: ‘Antibiotic Resistance Game’ (http://forio.com/simulate/busekirmaz92/antibiotic-resistance (1 May 2019, last accessed date)); ‘Stochastic Simulation of *E. coli* Antibiotic Resistance’ (https://github.com/KathyGCY/Antibiotic-Resistance (1 May 2019, last accessed date)); ‘Antibiotic Resistance Evolution Simulator’ (http://gydb.org/ares/public/index.php (1 May 2019, last accessed date)); or ‘Superbugs’ (https://longitudeprize.org/superbugs (1 May 2019, last accessed date)). These applications have one feature in common: the simulation of the effects of antibiotic resistance, in general. Some were aimed at being used to teach the concept of antibiotic resistance, but none allows for a great customization of the simulation, thus limiting the possibilities of engaging students in scientific practices.

Here, we present SimulATe—simulator of antibiotic therapy effects on the dynamics of bacterial populations—a computer program that simulates the effect of antibiotic therapies on bacterial populations and the role of antibiotic resistance on the sustainability of bacterial communities. The SimulATe application was developed with the following objectives in mind: (1) user-friendliness; (2) allow manipulation of several variables and parameters; (3) allow the testing of hypotheses and predictions and (4) adapt to different scenarios. These features make this software interesting from an educational point of view, providing teachers with a resource, which can help to foster their students’ scientific literacy and also make it an interesting tool with which to perform scientific research about antibiotic resistance evolution and its impact on bacterial communities.

## Materials and methods

SimulATe was written using the Python 2.7.13 [19] programming language. The Kivy framework 1.10.0 [20] and the Kivy module graph [21] were used to develop the graphical-user interface. To package the application into a runnable executable on Windows systems, the PyInstaller 3.2.1 [22] package was used. This software consists of two simulation scenarios [3], of which we chose to focus on the ‘Single Population’ scenario, for the purpose of this study. SimulATe is composed of a graphical-user interface which contains a ‘Flow Control’ section, a ‘Graph’ section, and a ‘Parameters and Options Configuration’ section. The ‘Flow Control’ section is a set of widgets that allows the user to start/pause/restart and control the speed of the simulation, the ‘Graph’ section is where the simulations run in real time and the ‘Parameters and Options Configuration’ section allows the user to set a variety of parameters and options, mostly directly associated with the equations described below.

### Equations

SimulATe makes use of several mathematical equations which, together, govern the interaction between antibiotic, bacteria and immune system. All equations were based on a study by Gjini and Brito [11]. Their differential equations were converted in difference equations, which are the discrete-time analogues of differential equations, using the Euler method. This is done as follows: Consider quantity X is governed by $\frac{dX}{dt}=f(X,\;\ldots )$. This is then changed towards $\frac{\Delta X}{\Delta t}=f(X,\;\ldots )=>\Delta X=f(X,\;\ldots )\cdot \Delta t$. But ΔX=X(t+Δt)-X(t). Therefore, the difference equation becomes: $X\left(t+\Delta t\right)-X\left(t\right)=f(X,\;\ldots )\cdot \Delta t$ or X(t+Δt) = X(t) + f(X, …)·Δt. The equations are the following:

Bacteria density (*B*)

(1) $$B\left(t+\Delta t\right)=B(t)+\left(\mathrm{rB}(t)-\mathrm{dB}(t)I-\delta B(t)\eta \left(t\right)A_{m}(t)\right)\cdot \Delta t.$$

This equation yields a new bacterial density for a given time step, where *d* is the time, *td* is the change in time, *r* is the growth rate of the bacteria, *d* is the rate at which lymphocytes inhibit the bacteria, *I* is the sum of all immune cells, *delta* is the rate at which antibiotic inhibits the bacteria (defines the difference between antibiotic resistant and susceptible bacteria), (*d*) indicates the presence of antibiotic, and $A_{m}(t)$ is the mean antibiotic concentration on the environment.

Naïve precursor cells density (*N*)

(2) $$N\left(t+\Delta t\right)=N(t)+\frac{-\sigma N(t)B(t)}{k+B(t)}\cdot \Delta t.$$

This equation yields a new naïve precursor cell density for a given time step, where *σ* is the maximum proliferation rate of the immune cells and *k* is the bacteria density at which the immune response grows at half its maximum rate, all other parameters are already defined in Equation (1).

Effector cells density (*E*)

(3) $$E\left(t+\Delta t\right)=E(t)+\left((2\sigma N(t)+\sigma E(t))\frac{B\left(t\right)}{k+B\left(t\right)}-\mathrm{hE}(t)\left(1-\frac{B(t)}{k+B(t)}\right)\right)\cdot \Delta t.$$

This equation yields a new effector cells density for a given time step, where h is the maximum decay rate of effector cells.

Memory cells density (*M*)

(4) $$M\left(t+\Delta t\right)=M\left(t\right)+\left(\mathrm{fE}\left(t\right)h\left(1-\frac{B\left(t\right)}{k+B\left(t\right)}\right)\right)\cdot \Delta t.$$

This equation yields new memory cells density for a given time step, where *f* is the fraction of effector cells which converts to memory cells.

Antibiotic uptake (*n*)

(5) $$\eta \left(t\right)\;=\;\{\begin{array}{c} 1\;\text{if}\;t_{1}<=t<=t_{1}\;+\;t_{2}\\ 0\;\text{if}\;t\;<\;t_{1}\;\text{or}\;t\;>\;t_{1}\;+\;t_{2} \end{array}.$$

For the classic treatment case, where *t*_1_ is the start of antibiotic treatment and *t*_2_ is the treatment duration, or

Antibiotic uptake (*n*)

(6) $$\eta \left(t\right)=\;\{\begin{array}{c} 1\;\mathrm{if}\;B\left(t\right)\geq \Omega \\ 0\;\mathrm{if}\;B(t)<\Omega \end{array},$$

for the adaptive treatment case, where *omega* is the defined bacteria density threshold. Both these equations yield the state of the antibiotic administration at each time step as a Boolean value, either 0 (antibiotic is being administered) or 1 (no antibiotic is being administered).

All parameters and default values used by the preceding equations are described in Table 1 in brief.

**Table 1:** parameters and default values

Symbol	Description	Default value	Range	Unit
*B*(0)	Initial antibiotic-sensitive bacterial density (*B*_s_)	10	1–100	cell/μl
	Initial antibiotic-resistant bacterial density (*B*_r_)	2		
*N*(0)	Initial naïve precursor cells density	200	0–1500	cell/μl
*E*(0)	Initial effector cells density	0	Fixed	cell/μl
*M*(0)	Initial memory cells density	0	Fixed	cell/μl
η(0)	Initial antibiotic uptake	0	0 or 1	–
*r*	Antibiotic-sensitive bacteria growth rate (*r*_s_)	3.3	0.1–8.0	day^−^¹
	Antibiotic-resistant bacteria growth rate (*r*_r_)	1.1	0.1–*r*_s_	
*d*	Bacteria lymphocyte inhibition	$10^{-5}$	$10^{-5}–10^{-4}$	μl/cell/day
*I*	Number of total immune cells	Varied	0–∞	cell/μl
δ	Antibiotic-sensitive bacteria antibiotic inhibition (δs)	1	0–1	l/mg/day
	Antibiotic-resistant bacteria antibiotic inhibition (δr)	0.1	0–*d*_s_	l/mg/day
$A_{m}$	Antibiotic mean concentration	6	1–120	mg/l
σ	Immune cells’ maximum proliferation rate	2	1.2–3.0	day^−^¹
*k*	Bacteria density at which the immune response grows at half its maximum rate	$10^{5}$	$10^{4}$–$10^{5}$	cell/μl
*h*	Effector cells’ maximum decay rate	0.35	0.1–0.8	day^−^¹
*f*	Fraction of effector cells which convert to memory cells	0.1	0.05–0.10	–
$t_{1}$	Start of antibiotic treatment	3.5	1–15	day
$t_{2}$	Treatment duration	7	3–15	day
Ω	Bacteria density threshold	$10^{6}$	$10^{3}$–$10^{7}$	cell/μl

Short description, default value, range and unit of every parameter used in the equations.

We chose this model as the basis of SimulATe because it takes into account the interaction between the host, pathogen, and antibiotic. The model makes some assumptions in regard to the workings of the immune system (Equations 2–4) reducing its complexity by reducing the number of immune cells and interactions between them. In the end, the immune cell algorithm is the following: naïve precursor cells (*N*, Equation 2) differentiate into effector cells (*E*, Equation 3) in response to increasing pathogen density. Effector cells proliferate as long as the pathogen density remains high. As the pathogen is cleared, most effector cells die except for a fraction that differentiates into memory cells (*M*, Equation 4) that persist for some time. Although these interactions are far from reality, they approximate very well the role the immune system has during a bacterial infection.

Most parameters preserve their default values and ranges from the original source study [11], but some were changed to allow for more realistic or broad ranged simulation scenarios and to make this software more interesting to educational purposes. The initial naïve precursor cells density range was then changed from 15-1500 to 0-1500 to allow the exclusion of the immune system from the simulation, this way scenarios without the effects of the immune system can be simulated, including non-human *in vitro* cultures or conditions. The range of the antibiotic mean concentration was also changed, from 0.03-128 to 1-120, to allow for better selection of a value in the user interface by removing the decimal values.

### Applying SimulATe as an educational resource

We developed two complementary problem-based learning (PBL) activities which can be explored using SimulATe as an educational resource (see Online Appendix 1). The first activity explores a scenario of antibiotic treatment interruption to foster students’ learning about evolution by natural selection, the role of the immune system in fighting antibiotic-resistant bacteria, and the applications of mathematical models in science as well as their limitations. The second activity explores the evolution of a bacterial community infecting an immunocompromised individual to foster learning about the immune system and the impacts of bacterial infections and antibiotic treatments in immunocompromised individuals. Together, these two activities explore scientific content that, in agreement with the official Portuguese program and guiding documents, should be learned by students in the 9th, 11th, and 12th grades [23–25]. But besides fostering scientific learning, the proposed tasks aim to simultaneously engage students in scientific practices and discussion which are fundamental for the development of their scientific literacy [17, 18] such as: asking questions; using models; planning and carrying out investigations; analysing and interpreting data; constructing explanations; arguing from evidence; and obtaining, evaluating, and communicating information.

Given the impacts of personal and medical decisions on the use of antibiotics, while using SimulATe as an educational resource, teachers should explore the importance, applications, and limitations of models in science. It should also be discussed under which circumstances these kinds of models can be used to inform public and private decisions. In agreement with NRC [18] recommendations, this discussion should help students learning that models are not copies of the reality and that researchers use models to make predictions about the likely outcomes of systems. It is also important that students understand that models are tested against other models and against observations from the reality, a process that can result in a revise of the initial model [18].

## Results and discussion

### Simulated dynamics of a bacterial infection

**Figure 1:**
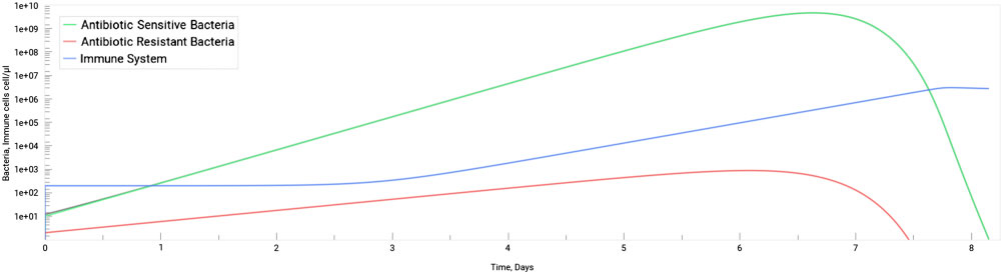
simulated dynamics of the infection by an arbitrary bacteria population, consisting of both antibiotic-sensitive (green) and antibiotic-resistant (red) individuals (the sum of both bacteria concentration in grey, although almost unnoticeable), and its interaction with the immune system (blue) in a virtual human microbiome. Values on the *x*-axis represent time measured in days since the beginning of the infection while values on the *y*-axis represent the density of both bacterial and immune system cells measured in cells/μl. To obtain this graph, parameters in the application were set to their default values and no treatment was selected (user treatment set to OFF).

The SimulATe software can be used to mimic the dynamics of a bacterial infection. We have performed a simulation of the population dynamics of a hypothetical single population bacterial infection triggered by the inoculation of a tissue by pathogenic bacteria (Fig. 1) using the default software parameters. As some bacteria can share the ability to code for antimicrobial resistance [represented in Equation (1) by the *delta* parameter], we assume that, in a bacterial population, some individuals will be able to persist in the presence of an antibiotic. In this scenario, we have considered antibiotic-sensitive bacteria as 10 times more abundant than resistant bacteria although growing at the same rate. These are the default parameters in SimulATe and will, from this point forwards, be considered as the standard condition. Given that bacteria reproduce by duplication, bacterial load will increase exponentially with time. In the human body, in an auto-limited bacterial infection, this growth will stimulate a pathogen-tailored immune response, proportional to the density of the pathogenic bacteria, which eventually clears the infection (Fig. 1), as can be seen in the simulation. However, pathogen’s ability to kill a human host is difficult to predict as it depends on the bacterial infectious dose, together with other factors such as, for example, pathogen’s infectiousness, its pathogenic power, or even on its ability to subvert the immune system. In humans, the infectious dose to trigger an infection can vary from three bacterial cells in the case of infections with *Orientia tsutsugamushi* to the high number of >10^10^ cells in infections with *Gardnerella vaginalis* [26]. The host may eventually die if the bacteria reach a very high density.

According to Gjini and Brito’s [11] model pathogen density causing symptoms is 10^3^–10^7^ cell/μl and it reaches 10^10^ cell/μl cells during infection in primary or unprotected infections. *In vivo* studies in murins, pathogenic bacterial load before the terminal stages of the infection can reach up to 10^8^–10^9^ CFU per ml or per organ: in lung infections by *Klebsiella pneumoniae*, *Staphylococcus aureus*, and *Pseudomonas aeruginosa* [27–29]; in the liver and spleen infections by *Listeria monocytogenes* and *Burkholderia pseudomallei* [30–32]; in the infection of the stomach by *Helicobacter pylori* [33]; or in infections related to orthopaedic devices, as with *S. aureus* [34]. These concentrations are all within the range allowed by SimulATe for varying the host death density parameter. Based on the information of these previously mentioned studies on pathogenic bacteria burden before terminal stages of infection, in this simulation, we have settled on a default threshold of 10^8^ cell/μl to define death in humans.

### The effect of early termination of antibiotic therapy

**Figure 2:**
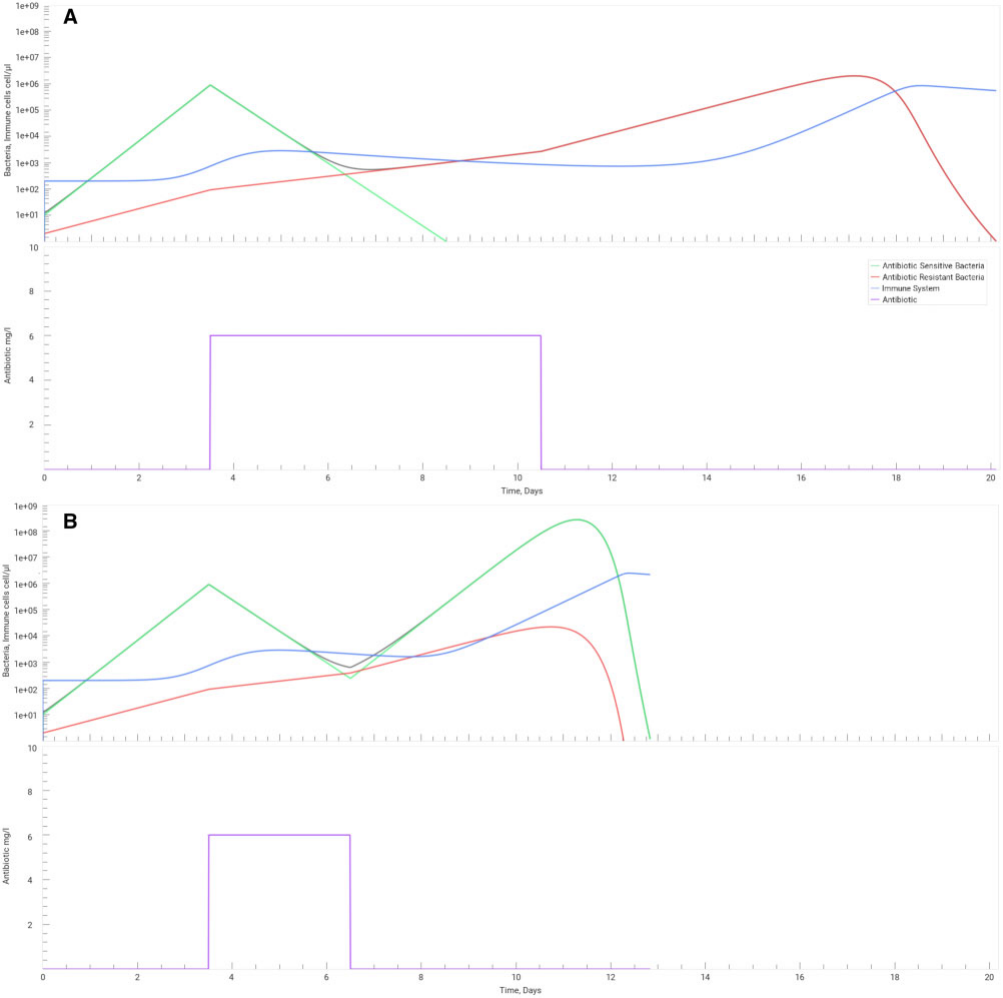
effects of the duration of the administration of an arbitrary antibiotic (purple) immune system (blue), antibiotic-sensitive (green) and antibiotic-resistant (red) bacteria (the sum of both bacteria concentration in grey). In both **A**) and **B**) the antibiotic is administered at a concentration of 6 mg/l starting at 3.5 days after infection. While in A) the treatment lasts for 7 days, resulting in a lighter infection, in B) the antibiotic therapy is interrupted earlier after just 3 days, exerting a much higher pressure on the immune system and reaching cell densities higher than those observed in A). To obtain these graphs, parameters in the application were set to their default values, the classical treatment was selected, and the duration parameter was set differently for each scenario (A = 7, B = 3). In all cases, the simulation stops when both bacteria reach a density of 0.

A recent survey conducted across 28 European countries reports that 15% of the respondents are convinced that they should stop taking antibiotic when symptoms disappear [6]. This makes early cessation of antibiotic therapy the most frequent case of antibiotic misuse. According to the ECDC an example of misuse of antibiotics is when the duration of antibiotic treatment is either too short or too long [7, 35]. In this context, we wanted to simulate a possible outcome of the shortening of the antibiotic treatment. As suggested in our first PBL activity, we used SimulATe to model the effects of a shorter antibiotic treatment on microbial load, as well as the proportion of sensitive and resistant bacteria under the effect of an antibiotic. For that we have generated a simulation of a standard treatment, according to the study developed by Gjini and Brito [11] (Fig. 2A), in which the antibiotic therapy lasted for 7 days, and we have compared it with a similar simulation but in which the treatment was interrupted at day 3 (Fig. 2B). In the first simulation (Fig. 2A), we can see a strong effect on the number of antibiotic-sensitive bacteria, which go extinct 9 days after the beginning of infection. The increasing number of bacteria induces an immune system response. Before the immune system is able to reach its maximum, a second peak of bacteria load is reached, this time consisting solely of antibiotic-resistant bacteria. Twenty days after infection, all resistant bacteria are cleared. In the second simulation (Fig. 2B), 3 days after the beginning of treatment, the bacterial load has diminished three orders of magnitude when compared with the beginning of the treatment or to the simulation of an untreated infection (Fig. 1). This can relieve symptoms and cause unaware patients to believe they are cured and lead them to terminate the antibiotic therapy earlier than expected and, possibly, store leftover antibiotics for a future situation, leading to self-medication. At this point, there are still 10^3^ sensitive bacteria that will enable bacterial growth past the infection threshold and, together with resistant bacteria, will enable a high pathogenic bacteria load and a potentially more severe relapse of the infectious disease. Bacterial clearance relies on the inducible effect of the immune system that is triggered by the higher bacterial load. In our simulation, we can see that although the symptoms are lighter, the high microbial burden can be potentially fatal to the host.

### The effect of a delayed start of antibiotic treatment

**Figure 3:**
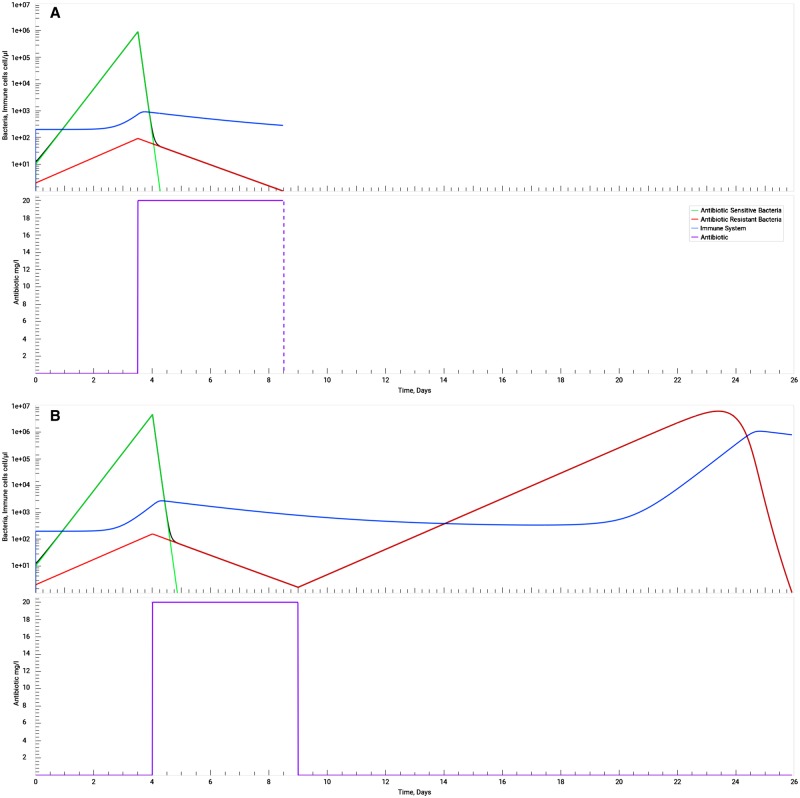
effect of the delayed start of the antibiotic therapy. Antibiotic-sensitive (green) and antibiotic-resistant (red) bacteria (the sum of both bacteria concentration in grey), immune system (blue), antibiotic (purple). In **A**) treatment starts at 3.5 days after infection and the duration of the treatment is just enough to eliminate the infecting bacteria completely (the full extent of the treatment is represented as a dashed line). In **B**) the treatment is delayed by just half a day and, with the same 5-day duration as in A), it is not enough to clear the infection and causes a resurgence of antibiotic-resistant bacteria a few weeks later, which is controlled by the immune system. To obtain these graphs, parameters in the application were set to their default values except for the antibiotic mean concentration, which was set at 20 mg/l. The classical treatment was selected with a duration of 5 days and the delay parameter was set differently for each scenario (A = 3.5, B = 4). In all cases, the simulation stops when both bacteria reach a density of 0.

According to ECDC, another example of antibiotic misuse consists on the delayed antibiotic administration in critically ill patients [7]. SimulATe can be used to study the expected outcomes of delayed antibiotic administration. Here, we present the simulation of one scenario where antibiotic therapy begins at two different times after infection: 3.5 days (Fig. 3A) and 4.0 days (Fig. 3B), both at a concentration of 20 mg/l. While comparing the two panels of Fig. 3, we can see that, in the case of the antibiotic therapy starting 3.5 days after infection, both bacteria were cleared after 5.0 days, i.e. 8.5 days after the beginning of the infection. On the other hand, when the patient delays the beginning of treatment for just half a day, the bacterial load reaches numbers of nearly an order of magnitude higher, causing a relapse of the disease. From there on, clearance would rely on the immune system, if the individual is immunocompetent. This simulation highlights the potential of SimulATe to perform deeper studies of the impacts of delayed antibiotic treatments, by simulating the expected outcomes across the parameters’ landscape.

### The use of antibiotics in immunosuppressed individuals

**Figure 4:**
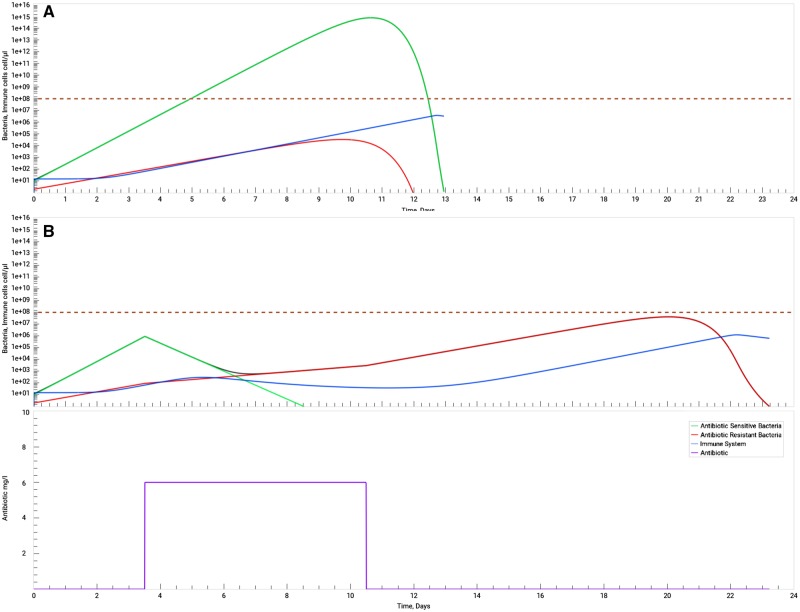
Administration of antibiotics in immunocompromised individuals. Antibiotic-sensitive (green) and antibiotic-resistant (red) bacteria (the sum of both bacteria concentration in grey), immune system (blue), antibiotic (purple). In **A**) a bacterial infection runs its course without the interference of an antibiotic to hinder its growth. As this specific individual has a compromised immune system, it is not able to subvert the bacterial development and dies 5 days after the initial infection (death threshold represented by a brown dashed line at a density of 10^8^ cell/μl). In **B**) an antibiotic therapy is applied, beginning 3.5 days after infection. In this case the individual is able to survive after the immune system, even though compromised, successfully eliminates the re-surging resistant bacteria. B) can be further compared with Fig. 2A, in which a normal immune system is in effect. To obtain these graphs, parameters in the application were set to their default values except for the immune system-related parameters, which were set as follows: initial precursor cell density = 15 cell/μl (min), proliferation rate = 1.2 day-1 (min), half maximum growth = 104 cell/μl (min), effector cell decay rate = 0.8 day-1 (max), memory cells conversion = 0.05 (min). Host death density was also set to 9.99e^14^ cell/μl (max) to prevent host death. While no treatment was selected for A) (user treatment set to OFF), in B) the classical treatment was selected with default parameters. In all cases, the simulation stops when both bacteria reach a density of 0.

The burden of a bacterial infection can be higher for individuals in fragile health conditions. The ECDC recommends that people over 65 years old, who suffer from chronic conditions such as asthma, diabetes, lung disease, or heart problems should seek medical help as soon as a bacterial infection is detected, rather than managing the symptoms without antibiotics [7]. Individuals with medical problems which cause the suppression of the immune system or under a therapy which suppresses the immune system (e.g. steroids, chemotherapy for cancer, and some drugs used to suppress thyroid gland functions) should be under special supervision. Immune system impairment reduces the individual’s ability to effectively eliminate the infecting bacteria [36]. We used the SimulATe software to model bacterial infections in individuals with immune system suppression or impairment as suggested in our second PBL activity. In fact, the simulated course of infection in these individuals (Fig. 4A) shows a dangerously higher peak of bacterial load (four orders of magnitude higher), as well as a longer duration of the infection when compared with the simulation of a healthy individual (Fig. 1). When we simulate the effect of a standard antibiotic treatment in an immunosuppressed individual (Fig. 4B) we can see that the immune cell response develops slower when compared with the simulation of an immune competent individual (Fig. 2A). This can be life threatening when under some bacterial infectious diseases. In the immunocompromised individual the infection clearance occurs nearly 24 days after the onset of the infection, i.e. it lasts nearly 9 days longer than in a healthy patient. Furthermore, if we consider the infecting bacteria as lethal at densities of 10^8^ cell/μl or higher, we can conclude that this individual would have died without the help of antibiotic therapy.

## Conclusion

Here, we present an application that was designed to: (1) be used for educational purposes; (2) be user-friendly, enabling the manipulation of several variables and parameters; (3) allow students to engage in several scientific practices required to become scientific proficient [17, 18]; and (4) allow the adaptation of teaching scenarios to different ages and levels.

Our results suggest that SimulATe cannot only model scenarios that support ECDC’s recommendations but also provide the required conditions for students to analyse the outcomes in distinct situation scenarios.

This also highlights the potential of using SimulATe for research projects that study the effects of antibiotics on bacterial population dynamics and to model realistic scenarios of treatment and its expected outcomes by varying different parameters of infectious processes. Nonetheless, a real parameterization could be difficult to achieve, which leads us to emphasize that the simulations should be assessed with caution and in its proper context.

One important limitation of the SimulATe software is that it does not take in account horizontal gene transfer of antibiotic resistance coding elements that shape population dynamics by changing the frequency of resistant bacteria in a population.

## Data availability

The SimulATe software is available, under the GPL-3.0 license, at https://github.com/Kronopt/SimulATe.

## Supplementary Material

bpz004_Supplementary_Data
