# Supplementary material for: Using SimulATe to model the effects of antibiotic selective pressure on the dynamics of pathogenic bacterial populations
Source: Biol Methods Protoc. 2019 May 29;4(1):bpz004. doi: 10.1093/biomethods/bpz004 (PMC7200973; doi:10.1093/biomethods/bpz004)
Supplement: bpz004_Supplementary_Data [file bpz004_supplementary_data.pdf]

Appendix 1

Using SimulATe to model the effects of antibiotic selective pressure on the dynamics of bacterial populations

Pedro HC David, Xana Sá-Pinto, Teresa Nogueira

The SimulATe software is available, under the GPL-3.0 license, at
https://github.com/Kronopt/SimulATe

Activity 1: SimulATing the consequences of patients' behaviours in their health

Target: high school and university students

Duration: 60 to 90 minutes

Learning goals: During this activity students are expected to learn about:

1) Evolution by natural selection;
2) The role of the immune system in fighting antibiotic resistant bacteria;
3) The value of scientific knowledge for our well-being;
4) The importance of making informed decisions in our daily life.

Students will also engage in the following scientific practices:

1) Ask questions;
2) Use mathematical models and discuss their applications and limitations;
3) Plan and carry out investigations;
4) Analyse and interpret data;
5) Constructing explanations;
6) Argue from evidence;
7) Obtain, evaluate and communicate information.

Activity 1: SimulATing the consequences of patients' behaviours in their health.

Last Monday, John's classmate had a fever in the morning and had to go home. The next day, John began to feel sick and had a fever at night. On Thursday, three days after his colleague spent the whole day coughing, John continued to feel sick. He went to the doctor, where he was diagnosed with a bacterial infection, and was prescribed an antibiotic that he should take for 8 days.
John began to take the antibiotic immediately and, three days later, he felt perfectly well. Then he began to wonder if he should or should not take the rest of the antibiotic. Although the doctor had stressed the importance of taking the antibiotic until the end, he had read in the newspaper (articles at https://www.theguardian.com/society/2017/jul/26/rule-patients-must-finish-antibiotics-course-wrong-study-says or https://www.dn.pt/sociedade/interior/estudo-defende-que-afinal-nao-e -preciso-tomar-antibioticos-até-fim-8667061.html) that some scientific studies suggested that antibiotic treatments should be discontinued as soon as patients showed no symptoms.

To help John understand the potential consequences of his choice, run the SimulATe program with the parameters corresponding to his infection and fill Table 1 with the expected results in each case.

Jonh's infection parameters: use **Default** options for all parameters except:

- Host Death Density: 10^8cell/ul
- Antibiotic treatment starting 3 days after the infection and lasting for 8 days.

Table 1 - Results of John's possible decisions

Decision	Relative frequency of resistant bacteria (cell/µl)				Outcome of Jonh's decision
	Start	3 days after	6 days after	8 days after	
Not taking any antibiotic					
Taking the antibiotic as prescribed by the doctor					
Treatment interrupted 3 days after its start					

Read the next questions, think about it, discuss it with your pair and then share your thoughts with the rest of the class:

1) What changes did you observe in the relative and absolute frequencies of resistant and non-resistant bacteria between the first and second days of infection? And between the 3rd and 6th days? What are the causes for the patterns you observe?

2) How do you explain the different outcomes of the three scenarios described in Table 1?

3) During the infection, what caused the reduction in the density of resistant bacteria?

4) Based on these results, what advices would you give to John?

5) Will these results always be reliable and match real cases? What may cause a discrepancy between the results obtained with SimulATe and the outcome of a real infection? And when do you think we can expect real results to more closely resemble those obtained with SimulATe?

Activity 2: John's grandfather visit

Target: high school and university students

Duration: 120 minutes

Learning goals: During this activity students are expected to learn about:

1) The circulatory system and its functions.
2) The immune system and the importance of effective antibiotic treatments for immunocompromised individuals;
3) The value of scientific knowledge for our well-being;
4) The importance of making informed decisions in our daily life.

Students will also engage in the following scientific practices:

1) Ask questions;
2) Use and discuss applications and limitations of mathematical models;
3) Plan and carry out investigations;
4) Analyse and interpret data;
5) Construct explanations;
6) Argue from evidence;
7) Obtain, evaluate and communicate information.

Activity 2: John's grandfather visit

On Thursday, on his way to John's place, his grandfather picked up the results from the blood test he had previously done.

Parameter	Concentration	Reference values
Red blood cells	$4.40X10^{12}/L$	$3,8 - 4.9\ X10^{12}/L$
Leukocytes	$1.4X10^{9}/L$	$4.0 - 11.0\ X10^{9}/L$
Platelets	$269X10^{9}/L$	$150 - 380\ X10^{9}/L$
Hemoglobin	13.8 g/dL	11.4 - 15.0 g/dL
Glucose	95 mg/dL	60 - 105 mg/dL
Urea	27 mg/dL	10 - 50 mg/dL
Uric acid	4.0 mg/dL	2.4 - 5.7 mg/dL
LDL-cholesterol	91 mg/dL	< 100 mg/dL
HDL-cholesterol	75 mg/dL	> 65 mg/dL
Insulin	20 U/ml	5 - 25 U/ml
Immunoglobulin E	70 kU/L	< 100 kU/L

Based on these results, investigate, think, discuss with your pair and share with the class:

1) Is there any evidence of health problems in John's grandfather blood test? Based on what you know, what advice would you give him?

2) Run SimulATe with the parameters corresponding to a possible infection in John's grandfather (see parameters below) and discuss your results with the class.

Jonh's grandfather infection parameters: use default options for all parameters except:

- Host Death Density: 10^8 cell/µl
- Initial precursor cell density: 106 cell/µl
- Proliferation rate: 1.2 per cell/µl/day
- Effector Cells Decay Rate: 0.8 cell/µl/day
- Memory Cells Conversion: 0.098
- Antibiotic treatment starting 3 days after infection and lasting 8 days

3) Imagine that John's grandfather doctor read the blood test results just after John was visited by his grandfather. What do you think this doctor can do to reduce the probability of bad consequences of John's grandfather visit to his grandson? Discuss with your pair, choose one hypothesis and test it with SimulATe.

**Experimental activity**	
**Our hipothesis**	
**How we are going to test our hypothesis**	
**Independent variable**	
**Dependent variables**	
**Results**	
**Conclusions**	

Additional sources of information

https://www.ted.com/talks/maryn_mckenna_what_do_we_do_when_antibiotics_don_t_work_any_more
https://www.youtube.com/watch?v=znnp-Ivj2ek

Additional activity:

✓ Organise a school campaign to promote the correct use of antibiotics. Do not forget to:
  - Clearly identify the important take home message;
  - Identify the communication channels that you will use, based on your colleagues' preferences and the message you want to communicate - be creative!
